# microRNAs Associated with Drought Response in the Bioenergy Crop Sugarcane (*Saccharum* spp.)

**DOI:** 10.1371/journal.pone.0046703

**Published:** 2012-10-11

**Authors:** Thaís Helena Ferreira, Agustina Gentile, Romel Duarte Vilela, Gustavo Gilson Lacerda Costa, Lara Isys Dias, Laurício Endres, Marcelo Menossi

**Affiliations:** 1 Laboratório de Genoma Funcional, Departamento de Genética, Evolução e Bioagentes, Instituto de Biologia, Universidade Estadual de Campinas. Campinas, São Paulo, Brazil; 2 Centro de Ciências Agrárias, Universidade Federal de Alagoas. Rio Largo, Alagoas, Brazil; 3 Laboratório Central de Tecnologias de alto desempenho, Universidades Estadual de Campinas. Campinas, São Paulo, Brazil; East Carolina University, United States of America

## Abstract

Sugarcane (*Saccharum* spp.) is one of the most important crops in the world. Drought stress is a major abiotic stress factor that significantly reduces sugarcane yields. However the gene network that mediates plant responses to water stress remains largely unknown in several crop species. Although several microRNAs that mediate post-transcriptional regulation during water stress have been described in other species, the role of the sugarcane microRNAs during drought stress has not been studied. The objective of this work was to identify sugarcane miRNAs that are differentially expressed under drought stress and to correlate this expression with the behavior of two sugarcane cultivars with different drought tolerances. The sugarcane cultivars RB867515 (higher drought tolerance) and RB855536 (lower drought tolerance) were cultivated in a greenhouse for three months and then subjected to drought for 2, 4, 6 or 8 days. By deep sequencing of small RNAs, we were able to identify 18 miRNA families. Among all of the miRNAs thus identified, seven were differentially expressed during drought. Six of these miRNAs were differentially expressed at two days of stress, and five miRNAs were differentially expressed at four days. The expression levels of five miRNAs (ssp-miR164, ssp-miR394, ssp-miR397, ssp-miR399-seq 1 and miR528) were validated by RT-qPCR (quantitative reverse transcriptase PCR). Six precursors and the targets of the differentially expressed miRNA were predicted using an *in silico* approach and validated by RT-qPCR; many of these targets may play important roles in drought tolerance. These findings constitute a significant increase in the number of identified miRNAs in sugarcane and contribute to the elucidation of the complex regulatory network that is activated by drought stress.

## Introduction

miRNAs are a class of small, non-coding RNAs, approximately 21 nucleotides in length, that are endogenous to both plants and animals [1], [2] and function to regulate gene expression by sequence-specific interaction with target mRNAs [3], [4]. In plants, each primary miRNA is transcribed by the RNA polymerase II enzyme [5] and forms an imperfect foldback structure that is processed into a stem-loop precursor (pre-miRNA) by Dicer-like 1 (DCL1), a nuclear RNase III-like enzyme. The pre-miRNA is then cleaved into a double-stranded RNA duplex, called miRNA/miRNA*, by the same enzyme [3], [6], [7]. One of the strands, called the mature miRNA, is incorporated into the RNA-induced silencing complex (RISC), whereas the other strand, called miRNA*, is usually degraded. The incorporated mature miRNA guides the RISC to a target mRNA by base pairing, leading to mRNA cleavage or translational repression [3], [4], [7].

Plants miRNAs were initially described in *Arabidopsis thaliana* [7] and since then, an increasing number of miRNAs have been identified in plants. miRNAs are known to be involved in developmental and physiological processes such as flowering, leaf and flower differentiation and the auxin response [2], [8]. Recently, the expression of a number of miRNAs has been found to be sensitive to abiotic and biotic stresses [9], [10], [11], [12], [13], [14], [15], with evidence that several play roles in drought stress [9], [16]. To date, 34 sugarcane miRNAs are reported (http://www.mirbase.org). Considering that drought stress significantly reduces sugarcane yields [17], the identification of sugarcane miRNAs that respond to this stress will help to elucidate the molecular basis of drought stress tolerance in this important bioenergy crop.

Deep sequencing strategies have revolutionized the discovery of small RNAs, constituting the most effective method for plant miRNA detection [18]. This strategy has allowed the discovery of species-specific and miRNAs that are expressed at very low levels [19], [20]. Because miRNAs are conserved across species, bioinformatics strategies [21], [22] based on expressed sequence tags (ESTs) from sugarcane and genomic survey sequences (GSSs) from sorghum were used in this study and homology and structural similarity. In this work, our goal was to identify and characterize miRNAs that may be regulated by water deficit in sugarcane.

## Results

### Molecular markers for drought in sugarcane plants

Two sugarcane cultivars differing in their tolerance to drought stress, RB867515 (higher tolerance, HT) and RB855536 (lower tolerance, LT), were grown in the greenhouse for three months. Water was withheld for 2, 4, 6 or 8 days, and stress symptoms, such as leaf rolling and senescence, appeared early on the second day. By the sixth day, almost all of the stressed plants were severely affected (Supporting Information, Figure S1 and Figure S2). Based on these results, we decided to evaluate the plants that were stressed at two and four days.

**Figure 1:**
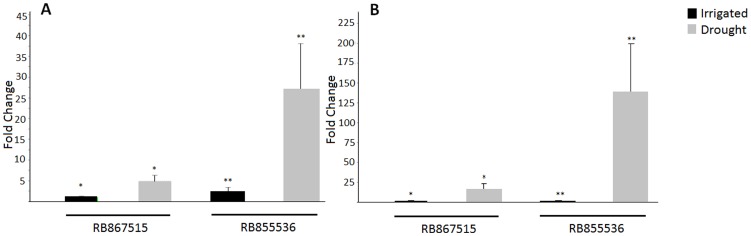
Real-time PCR of a sugarcane gene encoding a dehydrin (SCQGLR1085F11.g). RB867515 (higher drought tolerance) and RB855536 (lower drought tolerance) plants were irrigated (black bars) or subjected to drought stress by withholding irrigation (gray bars) for two (A) and four (B) days. Error bars represent the standard error (n = 4). * p<0.07 and ** p<0.05. Statistics was calculated between irrigated and drought treatments in each cultivar using the permutation mean test. The expression in irrigated RB867515 plants was considered as 1.

To characterize the degree of stress at the molecular level, we performed RT-qPCR (quantitative reverse transcriptase PCR) amplification of a sugarcane gene encoding a dehydrin (Fig. 1). The sugarcane assembled sequence (SAS) SCQGLR1085F11.g is induced by drought in sugarcane [23], and several homologues have been reported to be modulated by this stress in other species [24], [25], [26], [27]. This gene was induced after two and four days of stress (Fig. 1) in both sugarcane varieties. However, the cultivar with lower tolerance displayed greater induction at both experimental time points.

### Transcriptome analysis of small RNAs in sugarcane

To identify the miRNAs involved in drought stress in sugarcane, eight small RNA libraries from mature leaves were sequenced using the Solexa technology. These libraries were representative of plants stressed for two and four days and control plants from both sugarcane cultivars. A total of 90 million reads were obtained, ranging from 8 to 15 million reads per library (Table 1), similar to the available sequencing data for citrus, sorghum and maize [19], [28], [29]. All Solexa reads were aligned against the GenBank (http://www.ncbi.nlm.nih.gov/) and RFam (http://rfam.sanger.ac.uk/) databases to sort the reads into categories (Table 1). All of the sequences were then regarded as miRNAs for further analysis.

**Table 1:** Small RNA deep-sequencing data from sugarcane leaves.

Category	HTD2				HTD4			
	Unique RNAs	Percent (%)	Total RNAs	Percent (%)	Unique RNAs	Percent (%)	Total RNAs	Percent (%)
antisense exon	12,762	0.50	108,151	1.22	15,312	0.47	86,674	0.65
sense exon	76,415	2.98	651,383	7.34	139,293	4.24	814,958	6.10
miRNA	23,950	0.94	1,375,593	15.49	26,926	0.82	3,467,791	25.95
rRNA	58,355	2.28	925,787	10.43	63,945	1.95	866,844	6.49
siRNA	59,384	2.32	370,579	4.17	61,333	1.87	552,249	4.13
snRNA	2,094	0.08	7,456	0.08	4,056	0.12	39,774	0.30
snoRNA	780	0.03	1,516	0.02	1,487	0.05	4,854	0.04
tRNA	13,254	0.52	549,361	6.19	19,375	0.59	1,399,409	10.47
unannotated	2,313,810	90.35	4,888,685	55.06	2,951,339	89.90	6,128,310	45.87
total small RNAs	2,560,804		8,878,511		3,283,066		13,360,863	

Category	HTI2				HTI4			
	Unique RNAs	Percent (%)	Total RNAs	Percent (%)	Unique RNAs	Percent (%)	Total RNAs	Percent (%)
antisense exon	10,400	0.49	102,953	1.21	16,899	0.44	131,528	0.87
sense exon	77,102	3.66	831,951	9.78	76,252	2.00	628,631	4.18
miRNA	19,404	0.92	1,310,300	15.40	32,423	0.85	4,305,127	28.62
rRNA	65,322	3.10	1,214,350	14.28	61,239	1.60	861,743	5.73
siRNA	50,116	2.38	286,130	3.36	88,861	2.33	763,503	5.08
snRNA	1,904	0.09	6,942	0.08	2,382	0.06	7,433	0.05
snoRNA	878	0.04	1,897	0.02	809	0.02	1,766	0.01
tRNA	14,825	0.70	788,156	9.27	13,975	0.37	520,672	3.46
unannotated	1,867,650	88.61	3,963,113	46.59	3,528,379	92.34	7,819,918	51.99
total small RNAs	2,107,601		8,505,792		3,821,219		15,040,321	

Category	LTD2				LTD4			
	Unique RNAs	Percent (%)	Total RNAs	Percent (%)	Unique RNAs	Percent (%)	Total RNAs	Percent (%)
antisense exon	13,525	0.52	130,805	1.30	17,226	0.59	154,743	1.17
sense exon	96,298	3.72	1,044,692	10.38	76,303	2.62	735,969	5.58
miRNA	22,271	0.86	1,501,828	14.93	22,744	0.78	3,483,293	26.42
rRNA	77,634	3.00	1,521,502	15.12	60,765	2.09	926,250	7.03
siRNA	58,478	2.26	399,760	3.97	63,817	2.19	587,248	4.45
snRNA	2,755	0.11	11,421	0.11	2,464	0.08	8,252	0.06
snoRNA	1,179	0.05	2,660	0.03	757	0.03	2,020	0.02
tRNA	17,741	0.69	797,948	7.93	14,831	0.51	727,495	5.52
unannotated	2,298,296	88.80	4,649,978	46.22	2,652,688	91.11	6,557,000	49.74
total small RNAs	2,588,177		10,060,594		2,911,595		13,182,270	

Category	LTI2				LTI4			
	Unique RNAs	Percent (%)	Total RNAs	Percent (%)	Unique RNAs	Percent (%)	Total RNAs	Percent (%)
antisense exon	14,157	0.64	131,400	1.40	14,757	0.55	161,302	1.33
sense exon	81,911	3.72	792,014	8.44	67,798	2.52	506,603	4.17
miRNA	20,041	0.91	1,515,240	16.15	23,936	0.89	3,682,698	30.32
rRNA	65,089	2.96	1,150,319	12.26	54,672	2.03	676,834	5.57
siRNA	63,279	2.87	469,568	5.00	68,251	2.53	664,751	5.47
snRNA	2,849	0.13	13,901	0.15	2,362	0.09	8,234	0.07
snoRNA	1,180	0.05	3,190	0.03	973	0.04	2,283	0.02
tRNA	14,087	0.64	736,610	7.85	13,576	0.50	415,579	3.42
unannotated	1,939,448	88.08	4,571,452	48.72	2,447,018	90.85	6,028,355	49.63
total small RNAs	2,202,041		9,383,694		2,693,343		12,146,639	

HTD: RB867515 (higher drought tolerance) plants under drought stress (without watering). HTI: RB867515 plants under irrigation. LTD: RB855536 (lower drought tolerance) under drought stress. LTI: RB855536 (lower drought tolerance) plants under irrigation. 2: two days of treatment. 4: four days of treatment.

**Figure 2:**
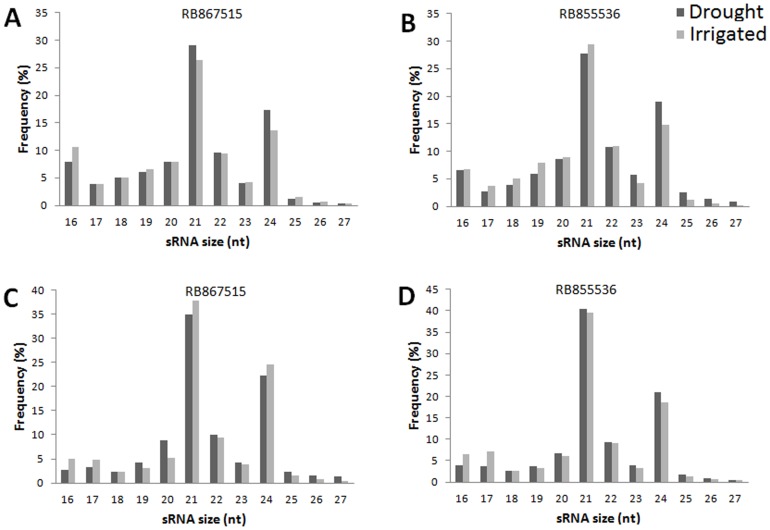
Size distribution of small RNA (sRNA) redundant sequences in two sugarcane cultivars. Cultivars RB867515 (A and C) and RB855536 (B and D) were irrigated (gray bars) or drought-stressed (black bars) for two (A and B) and four (C and D) days. RB867515 is the higher drought tolerant genotype, and RB855536 is the lower drought tolerant genotype.

**Figure 3:**
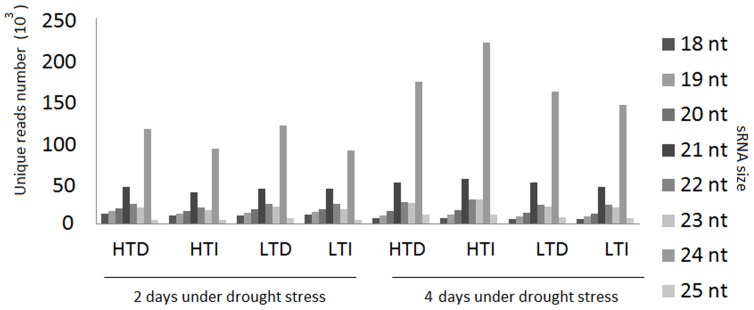
Size distribution of small RNA (sRNA) non-redundant sequences in two sugarcane cultivars. HTD: RB867515 (higher tolerance cultivar) plants without watering. HTI: RB867515 plants under irrigated conditions. LTD: RB855536 (lower tolerance cultivar) plants without watering. LTI: RB855536 plants under irrigated conditions.

**Figure 4:**
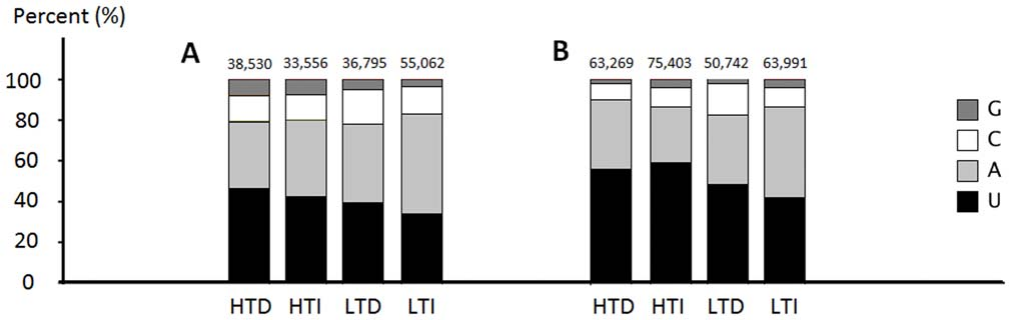
Composition of nucleotides in the first base positions of all 21 nt sequences. A: Control and stressed plants after two days of drought conditions. B: Control and stressed plants after four days of drought conditions. HTD: RB867515 (higher tolerance cultivar) plants without watering. HTI: RB867515 (higher tolerance cultivar) plants under irrigated conditions. LTD: RB855536 (lower tolerance cultivar) plants without watering. LTI: RB855536 (lower tolerance cultivar) plants under irrigated conditions. The total numbers of 21 nt reads in each library are shown at the tops of the bars.

The size distribution of all of the sequences is presented in Fig. 2. The majority of the reads were 21 to 24 nt in length for all libraries, with 21 nt being the most redundant species, followed by 24 nt (Fig. 2). However, sequences of 24 nt were the most represented class of non-redundant species (Fig. 3). In the 21 nt reads, approximately 80% was composed of U or A at the first base position at the 5′end in all libraries (Fig. 4), with equal number of each nucleotide.

### Identification of new potential miRNAs in *Saccharum* spp

To identify sugarcane miRNA candidates within our sequenced set, unique small RNA species were aligned against the *Sorghum bicolor* genome to identify loci corresponding to putative miRNA precursors. A total of 21 miRNAs were detected corresponding to 18 miRNAs families in sugarcane: miR156, miR160, miR164, miR166, miR167, miR169, miR171, miR172, miR319, miR390, miR393, miR394, miR396, miR397, miR399, miR528, miR529 and miR1432 (Table 2). The sugarcane miRNAs were named based on their homology to sorghum miRNAs.

**Table 2:** Sugarcane miRNAs identified by Solexa sequencing.

miRNA family	miRNA	mature sequence	Sorghum precursor
miR1432	ssp-miR1432	UCAGGAAAGAUGACACCAA	sbi-MIR1432
miR156	ssp-miR156	UUGACAGAAGAGAGUGAGCAC	sbi-MIR156b
			sbi-MIR156c
			sbi-MIR156d
			sbi-MIR156e
			sbi-MIR156g
			sbi-MIR156h
			sbi-MIR156i
miR160	ssp-miR160seq1	UGCCUGGCUCCCUGUAUGCCA	sbi-MIR160c
miR164	ssp-miR164	UGGAGAAGCAGGGCACGUGCA	sbi-MIR164b
miR166	ssp-miR166seq1	UCGGACCAGGCUUCAUUCCCC	sbi-MIR166a
			sbi-MIR166b
			sbi-MIR166c
			sbi-MIR166d
			sbi-MIR166h
			sbi-MIR166j
miR167	ssp-miR167b	UGAAGCUGCCAGCAUGAUCUGA	sbi-MIR167c
			sbi-MIR167d
			sbi-MIR167e
			sbi-MIR167g
			sbi-MIR167h
			sbi-MIR167i
miR169	ssp-miR169seq1	CUAGCCAAGAAUGACUUGCCU	sbi-MIR169f
	ssp-miR169seq2	CAGCCAAGGAUGACUUGCCGA	sbi-MIR169a
miR171	ssp-miR171seq1	UUGAGCCGCGUCAAUAUCUCC	sbi-MIR171h
	ssp-miR171seq2	UGAUUGAGCCGUGCCAAUAUC	sbi-MIR171g
			sbi-MIR171i
			sbi-MIR171k
	ssp-miR171seq3	UGAGCCGAACCAAUAUCACUC	sbi-MIR171c
			sbi-MIR171f
miR172	ssp-miR172	AGAAUCUUGAUGAUGCUGCAU	sbi-MIR172a
			sbi-MIR172d
			sbi-MIR172e
miR319	ssp-miR319	UUUGGAUUGAAGGGUGCU	sbi-MIR319b
miR390	ssp-miR390	AAGCUCAGGAGGGAUAGCGCC	sbi-MIR390
miR393	ssp-miR393	CUCCAAAGGGAUCGCAUUGAU	sbi-MIR393b
miR394	ssp-miR394	UUGGCAUUCUGUCCACCUCC	sbi-MIR394a
miR396	ssp-miR396seq1	UCCACAGGCUUUCUUGAACUG	sbi-MIR396d
miR397	ssp-miR397	UUGACUGCAGCGUUGAUGAGC	sbi-MIR397
miR399	ssp-miR399seq1	UGCCAAAGGAGAGUUGCCCUG	sbi-MIR399a
			sbi-MIR399f
			sbi-MIR399h
			sbi-MIR399i
			sbi-MIR399j
miR528	ssp-miR528	UGGAAGGGGCAUGCAGAGGAG	sbi-MIR528
miR529	ssp-miR529	AGAAGAGAGAGAGUACAGCCU	sbi-MIR529

miRNAs found in the leaves of two sugarcane cultivars (RB867515 and RB855536). Two mismatches were allowed against sorghum mature miRNAs.

**Figure 5:**
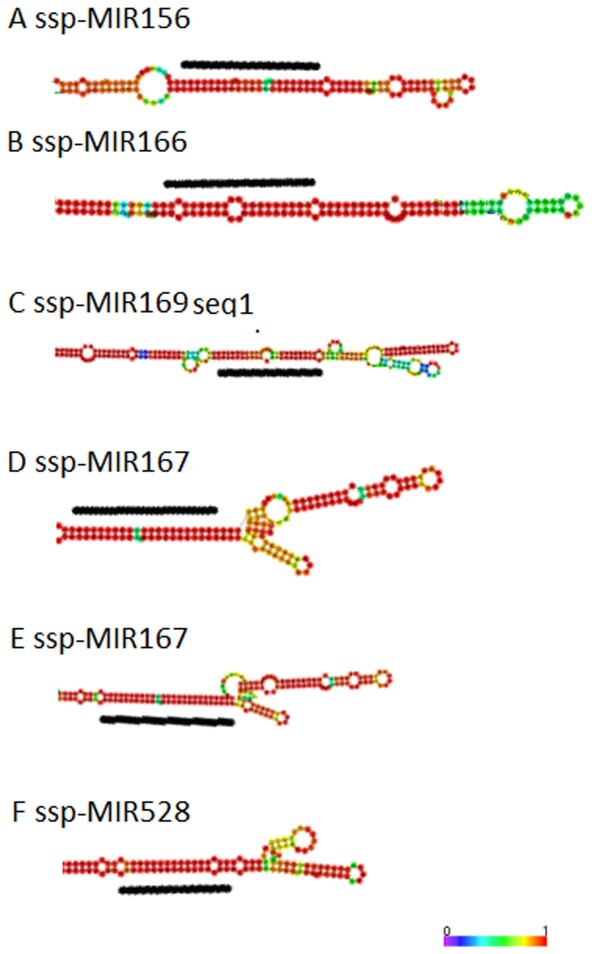
Primary transcripts containing the predicted stem-loop structures of the precursors of the sugarcane miRNAs. The mature miRNAs identified in the sugarcane sRNA library are highlighted in black. The sizes of the precursors may be slightly longer than represented. The colors represent the probabilities for sequence alignment. Red is the highest probability of alignment (1), and purple is the lowest probability of alignment (0).

Precursors were also identified among the sugarcane ESTs (Table 3 and Fig. 5) derived from six genomic loci because six different SASs were found to correspond to six different miRNA genes (Table 3) and their RNA sequences have the intramolecular capacity to fold into hairpin structures (Fig. 5). Five precursor sequences were from the SUCEST (http://sucest-fun.org/) database, whereas only one was found in the SoGI (http://compbio.dfci.harvard.edu/) database (Table 3). Two distinct SASs were found for the ssp-miR167 precursor. The MFEs (minimum free energy) ranged from −145 to −338 kcal/mol, and the G/C content ranged from 42 to 49% (Table 3).

**Table 3:** Bioinformatics prediction of sugarcane miRNAs precursors.

Precursor name	Cluster	MFE (Kcal/mol)	G/C (%)	Mature miRNA sequence (5′ - 3′)
ssp-MIR156	SCSBAD1086B12	−155	43	UUGACAGAAGAGAGTGAGCAC
ssp-MIR166	TC144774	−145.32	45	UCGGACCAGGCUUCAUUCCCC
ssp-MIR167a	SCMCSD2060C04	−156.92	46	UGAAGCUGCCAGCAUGAUCUGA
ssp-MIR167b	SCSFSD1065B12	−225.44	42	UGAAGCUGCCAGCAUGAUCUGA
ssp-MIR169d	SCJLRZ1019E10	−338.87	48	CAGCCAAGGATGACTTGCCGA
ssp-MIR528	SCUTSD1026H02	−204.5	49	UGGAAGGGGCAUGCAGAGGAG

The corresponding precursor clusters in SOGI and SAS in the SUCEST databases are indicated in the Cluster column. MFE: minimum free folding energy. G/C contents and the mature miRNA sequences are also shown.

### Analysis of sugarcane miRNAs modulated by drought stress

**Figure 6:**
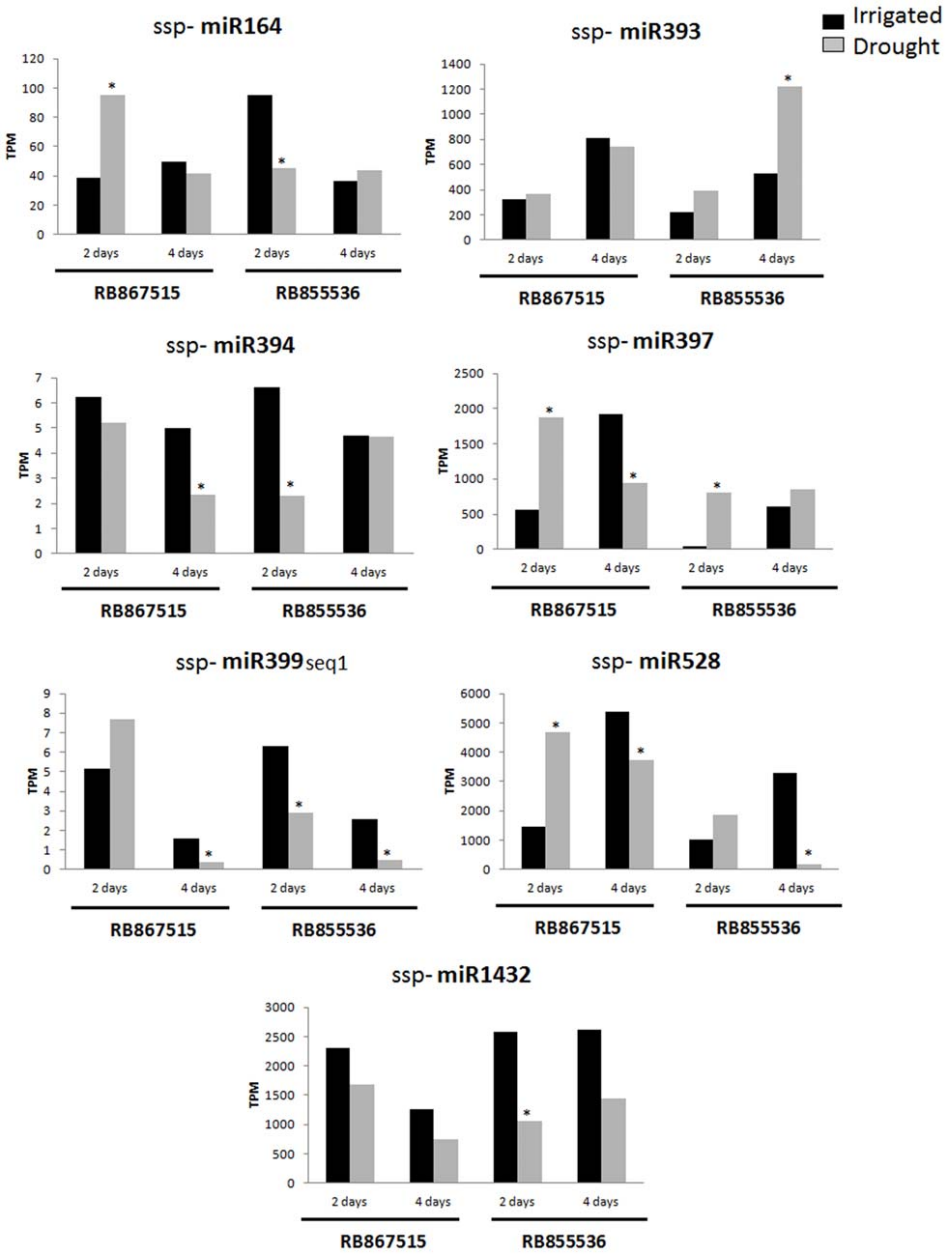
Expression profiles of seven sugarcane miRNAs modulated by drought stress. The values are expressed as the number of transcripts per million (TPM) for irrigated plants (control, black bars) and drought-stressed plants (gray bars). RB867515 (higher drought tolerance) and RB855536 (lower drought tolerance) plants were evaluated after two and four days of stress, as indicated below each graphic. * p<0.05 and fold change >2.0. Statistics was calculated between irrigated and drought treatments in each cultivar using the Audic-Claverie method [78].

Among all of the miRNAs identified, seven were differentially expressed during drought (Fig. 6), of which six were differentially expressed after two days of stress (Table 4) and five were differentially expressed after four days of stress (Table 5). The ssp-miR164 miRNA was differentially expressed only at two days of stress. Additionally, three miRNAs (ssp-miR164, ssp-miR397 and ssp-miR528) were up-regulated in the RB867515 (higher tolerance, HT) cultivar, and none were down-regulated by drought. In the RB855536 (lower tolerance, LT) cultivar, four miRNAs (ssp-miR164, ssp-miR394, ssp-miR399-seq 1 and ssp-miR1432) were down-regulated, and only one (ssp-miR397) was up-regulated by drought. Only ssp-miR397 displayed the same pattern in both cultivars, as it was induced after two days of water stress (Table 4).

**Table 4:** Sugarcane miRNAs differentially expressed after two days of drought stress.

Family	miR name	Mature sequences	Nt	HTD (TPM)	HTI(TPM)	LTD(TPM)	LTI (TPM)	HTD/HTI	LTD/LTI
miR164	ssp-miR164	UGGAGAAGCAGGGCACGUGCA	21	95.07	38.57	45.43	95.28	2.46*	−2.10*
miR394	ssp-miR394	UUGGCAUUCUGUCCACCUCC	20	5.19	6.24	2.30	6.62	−1.20	−2.88*
miR397	ssp-miR397	UUGACUGCAGCGUUGAUGAGC	21	1873.63	562.10	799.76	42.42	3.33*	18.85*
miR399	ssp-miR399seq1	UGCCAAAGGAGAGUUGCCC	19	7.67	5.18	2.89	6.30	1.48	−2.18*
miR528	ssp-miR528	UGGAAGGGGCAUGCAGAGGAG	21	4674.10	1445.14	1874.15	1025.19	3.23*	1.83
miR1432	ssp-miR1432	UCAGGAAAGAUGACACCAA	19	1687.91	2300.44	1053.92	2583.96	−1.36	−2.45*

Nt: number of nucleotides of the mature miRNA. HTD: RB867515 (higher drought tolerance) plants under drought stress (without watering). HTI: RB867515 plants under irrigation. LTD: RB855536 (lower drought tolerance) under drought stress. LTI: RB855536 (lower drought tolerance) plants under irrigation. In the last two columns, negative values indicate down-regulated miRNAs and positive values indicate miRNAs that are up-regulated during drought. Asterisks indicate minimum fold change >2.00 and p<0.05.

**Table 5:** Sugarcane miRNAs differentially expressed after four days of drought stress.

Family	miR name	Mature sequences	Nt	HTD(TPM)	HTI(TPM)	LTD(TPM)	LTI(TPM)	HTD/HTI	LTD/LTI
miR393	ssp-miR393	CUCCAAAGGGAUCGCAUUGAU	21	743.15	808.97	1220.51	582.06	−1.09	2.10*
miR394	ssp-miR394	UUGGCAUUCUGUCCACCUCC	20	2.33	5.00	4.64	4.70	−2.14*	−1.01
miR397	ssp-miR397	UUGACUGCAGCGUUGAUGAGC	21	940.44	1926.63	858.82	602.07	−2.05*	1.43
miR399	ssp-miR399seq1	UGCCAAAGGAGAGUUGCCC	19	0.38	1.61	0.47	2.56	−4.18*	−5.51*
miR528	ssp-miR528	UGGAAGGGGCAUGCAGAGGAG	21	3717.14	5397.83	182.68	3293.76	−1.45*	−18.03*

Nt: number of nucleotides of the mature miRNA. HTD: RB867515 (higher drought tolerance) plants under drought stress (without watering). HTI: RB867515 plants under irrigation. LTD: RB855536 (lower drought tolerance) under drought stress. LTI: RB855536 (lower drought tolerance) plants under irrigation. In the last two columns, negative values indicate down-regulated miRNAs and positive values indicate miRNAs that are up-regulated during drought. Asterisks indicate minimum fold change >2.00 and p<0.05.

ssp-miR393 was only differentially expressed at four days of stress. Four miRNAs (ssp-miR394, ssp-miR397, ssp-miR399-seq 1 and ssp-miR528) were down-regulated by drought in the HT cultivar. Two other miRNAs (ssp-miR399-seq 1 and ssp-miR528) were down-regulated, and one (ssp-miR393) was up-regulated in the LT cultivar. Only miRNAs ssp-miR399-seq 1 and ssp-miR528 were down-regulated in both cultivars (Table 5).

Four miRNAs (ssp-miR394, ssp-miR397, ssp-miR399-seq 1 and miR528) were differentially expressed at both time points during the stress period, and only one miRNAs (ssp-miR399-seq 1) presented similar expression profiles in the LT cultivar (Table 4 and 5).

**Figure 7:**
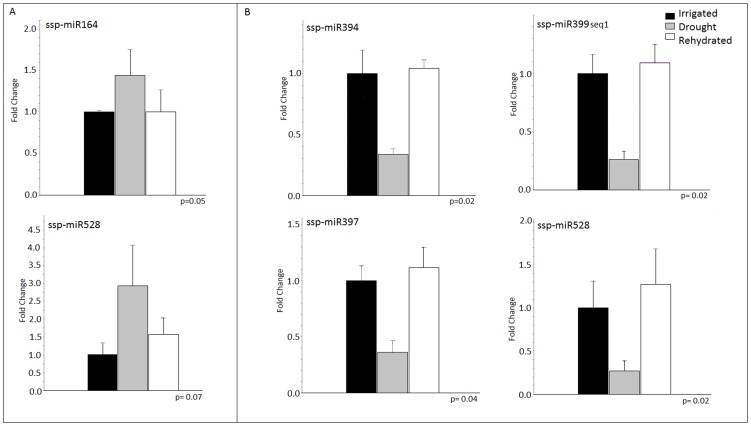
RT-qPCR expression profiles of five sugarcane miRNAs modulated by drought stress. The values are expressed as fold changes relative to the irrigated control for each gene. The bars represent the average of the irrigated plants (control, black bars), drought-stressed plants (gray bars) and rehydrated plants (white bars) for RB867515 (higher drought tolerance) after two (A) and four (B) days of stress. Error bars represent the standard error (n = 4). Means followed by different letters are statistically different (p<0.05) using the permutation mean test.

Stem-loop RT-qPCR [30], [31] was used to validate the expression of all seven differentially expressed miRNAs during drought according to the sequencing data. The expression patterns of five miRNAs (ssp-miR164, ssp-miR394, ssp-miR397, ssp-miR399-seq 1 and ssp-miR528) were validated by this approach. Among the 15 differentially expressed profiles observed with the sequencing approach (Fig. 6), five (33.33%) displayed the same expression profile using RT-qPCR (Fig. 7). The RT-qPCR analysis confirmed two out of eight (25%) profiles after two days of stress and four out of seven (57%) after four days. In the HT cultivar, five out of seven (71.4%) profiles were confirmed, whereas in the LT cultivar, no profile was confirmed. In all of the RT-qPCR profiles, the miRNA levels of the rehydrated plants demonstrated a tendency to return to the control levels (Fig. 7).

### Prediction of miRNA targets

The mature sequences of the seven miRNAs modulated by drought were used to search for their targets in sugarcane (Table 6 and Supporting information, Table S1). All of these miRNAs had putative targets in the SUCEST database (Table 6), several of which encode transcription factors (ssp-miR164, ssp-miR394, ssp-miR528 and ssp-miR1432), growth or development regulators (ssp-miR393), proteins associated with floral development (ssp-miR394) and several phosphatases, kinases, and oxidases (ssp-miR394, ssp-miR397, ssp-miR399-seq 1, ssp-miR528 and ssp-miR1432), among others (Supporting Information, Table S1). The targets had homologous proteins in several plant species, especially maize, rice and *Brachypodium*, suggesting that the pathways where these targets act may be conserved.

**Table 6:** Target prediction of the miRNAs that are differentially expressed in drought-stressed sugarcane plants.

miRNA	Target Acc.	Expectation	UPE	mature miRNA	Target start	Target end	miRNA aligned fragment	Target aligned fragment	Inhibition	Target description
ssp-miR164	SCEPRT2048G05.g	1.0	20,052	20	699	718	UGGAGAAGCAGGGCACGUGC	GCAGGUGCCCUGCUUCUCCA	Cleavage	NAC transcription factor [*Hordeum vulgare*]
	SCCCAM1001A03.g	2.5	20,361	19	1774	1792	UGGAGAAGCAGGGCACGUG	CACGUGUCCACCUUCUCCA	Translation	MDR-like ABC transporter [*Oryza sativa Japonica Group*]
ssp-miR394	SCQGAM2027G09.g	3.0	19,627	19	759	777	UUGGCAUUCUGUCCACCUC	UUGGCAUUCUGUCCACCUC	Cleavage	Glyceraldehyde-3-phosphate dehydrogenase [*Triticum aestivum*]
ssp-miR528	SCJFRT2058D11.g	2.5	22,413	20	101	120	UGGAAGGGGCAUGCAGAGGA	UUCUCGGCAUGCCCCUUCUG	Cleavage	UBX domain-containing protein [*Oryza brachyantha*]
ssp-miR397	SCQSAD1056B07.g	2.5	22,911	20	64	83	UUGACUGCAGCGUUGAUGAG	UUCAUCAACGCCGCACUCAA	Translation	laccase-23-like [*Brachypodium distachyon*]
ssp-miR1432	TC134052	3.0	17.32	19	630	648	UCAGGAAAGAUGACACCAA	UUGGUGUUUUCUUCCCUGA	Translation	ABRE-binding factor BZ-1 [*Zea mays*]
	SCSFFL4085D03.g	3.0	15,088	19	624	642	UCAGGAAAGAUGACACCAA	UUGGUGUUUUCUUCCCUGA	Translation	bZIP transcription factor1 [*Zea mays*]
ssp-miR393	TC120009	1.0	20,653	19	302	320	CUCCAAAGGGAUCGCAUUG	CAAUGCGAUCCCUUUGGAU	Cleavage	Auxin-responsive factor TIR1-like protein [*Populus tomentosa*]
ssp-miR399seq1	SCACHR1037A06.g	1.5	19,642	19	463	481	UGCCAAAGGAGAGUUGCCC	GGGUAGUUCUCCUUUGGCA	Cleavage	inorganic pyrophosphatase 2-like [*Brachypodium distachyon*]

Target Acc: accession number in the SUCEST or SoGI databases; Expectation: value assigned to the alignment of the mature miRNA and the target, ranging from 0 (perfect alignment) to 5; UPE: the energy required to open the secondary structure of the target at the recognition site (less energy means better accessibility to the target); Mature miRNA: miRNA mature size (in nucleotides); Target start: the base position where the annealing with the miRNA starts; Target end: the base position where the annealing with the miRNA ends; Inhibition: the type of regulation by the miRNA; and Target description: description of the target according to a BLAST search in the GenBank database, including the name of the organism presenting the best hit.

### Target validation by RT-qPCR

RT-qPCR was used to validate the expression of seven putative target genes, one for each of the miRNAs modulated by drought (Supporting Information, Figure S3). To correlate the expression profiles of miRNAs and target genes we calculated the expression ratios between drought-stressed and control plants (Table 7). Considering the 15 treatments where miRNA expression were statistically significant (P<0.05, Fig. 6), we found that in seven treatments target genes had the expected profile, i.e., induced expression of miRNA and repressed expression of the target gene and vice-versa (Table 7, underlined ratios). In two out the seven target profiles, RT-qPCR expression was also statistically significant at P<0.05. This data suggest that in several cases sugarcane miRNA might regulate target genes by translational repression and not by mRNA degradation.

**Table 7:** Validation of the target genes found for the miRNAs differentially expressed under drought.

miRNA Target	SAS/SoGI cluster name	Target description	HT2	HT4	LT2	LT4
target miR164	SCEPRT2048G05.g	NAC transcription factor	2.5*/1.2	**0.8/3.4**	**0.5** * **/3.9**	1.2/2.5*
target miR393	TC120009	Auxin-responsive factor TIR1-like protein	1.1/0.6*	0.9/0.3	1.7/0.9	2.3*/2.0
target miR394	SCQGAM2027G09.g	Glyceraldehyde 3-Phosphate Dehydrogenase	0.8/0.7	0.5*/0.6	0.3*/1.0	1.0/1.7
target miR397	SCQSAD1056B07.g	Lacasse 23-Like	3.3*/1.1	**0.5** * **/6.9** *	**18.9** * **/0.7**	1.4/11.5
target miR399	SCACHR1037A06.g	Inorganic pyrophosphatase 2-like	1.5/2.3	0.2*/0.1	**0.5** * **/1.8**	0.2*/0.5
target miR528	SCJFRT2058D11.g	UBX Domain Containing Protein	**3.2** * **/0.4** *	0.7*/0.4*	**1.8/0.5** *	**0.1** * **/2.0**
target miR1432	SCSFFL4085D03.g	B-ZIP transcription factor	**0.7/4.0** *	**0.6/4.1**	**0.4** * **/2.0**	**0.5/11.7** *

The expression rations between drought-stressed and control plants are shown. The first number in each pair indicates miRNA levels and the second indicates the target gene expression.

* indicates ratios where differences in the expression levels between irrigated and drought-stressed plants are statistically significant (P<0.05). Expression rations that are in bold indicate that miRNA induction or repression correlates with repression or induction of target genes, respectively. SAS: Sugarcane Assembled Sequence; SoGI: Sugarcane Gene Index; HT: higher tolerance cultivar; LT: lower tolerance cultivar; 2: two days of stress; 4: four days of stress.

## Discussion

### The sugarcane microRNAs

Although the miRNAs of several plant species have been recently studied, neither miRNA sequence identification nor an analysis of differential miRNA expression in response to drought stress has been performed in sugarcane. We identified 21 sugarcane miRNAs comprising 18 families using deep sequencing (Table 2). Because the sugarcane genome has not yet been sequenced, *Sorghum bicolor* was chosen as a reference organism because of the high level of identity between the genes in both species [32], [33], [34]. One of the strategies for the characterization of miRNAs is to examine the phylogenetic conservation of their sequences [35], which permitted the identification and classification of the miRNAs from eight sugarcane libraries. The identification of these miRNAs was accomplished by precursor sequence folding into genuine hairpin structures of the *S. bicolor* sequences where the reads matched, and their classification was based on homology to *S. bicolor* miRNAs in the miRBase database.

In this study, only six putative precursors were found in the sugarcane EST databases, representing five miRNA families (Fig. 5 and Table 3). Because such precursors have short lifetimes in plants due to rapid processing by Dicer-like 1 in the nucleus [3], [36], EST databases do not typically have sufficient coverage to facilitate the discovery of a great number of precursors. For example, in *Brachypodium distachyon*, a model organism for grass species, only 0.05% of the ESTs and 0.012% of the GSSs contain potential miRNAs [37]. The five families found in sugarcane have been previously described, and the secondary structures of the pre-miRNAs have been evaluated using an *in silico* approach [38]. The majority of the small RNAs in sugarcane are 21 or 24 nt in length, as expected in plants (Fig. 2) [4]. The 24 nt species are the most abundant in the non-redundant pool of short reads in sugarcane (Fig. 3), and similar distributions have been reported in studies with several other organisms [18], [39], [40]. This distribution is likely because 24 nt RNAs are siRNAs and unlike miRNAs, a longer double strand of RNA can result in several different small sequences that act in post-transcriptional gene silencing (PTGS).

It has been reported that uracil appears to be dominant as the first nucleotide at the 5′ ends of mature miRNAs [41]. In sugarcane, the first positions of the mature sequences were equally composed of U and A (Fig. 4). The 5′end nucleotide of a small RNA strand determines the identity of at least several of the recruited AGO proteins [42]. Thus, the 5′end nucleotide has important implications for miRNA function because AGO1 preferentially associates with 21 nt RNAs with a U at the 5′ end, resulting in PTGS activity. In contrast, AGO2 seems to preferentially associate with 21 nt RNAs that have an A at the 5′ end, abolishing their silencing activity and rendering the miRNA functionally inert [43].

### miRNAs associated with drought response in sugarcane

To identify miRNAs that are differentially expressed during drought, eight libraries from sugarcane leaf were analyzed: four from RB867515, a cultivar with high tolerance to drought, and four from RB855536, which has a lower drought tolerance. A total of seven miRNAs were differentially expressed during drought (Fig. 6). Six of these were differentially expressed at two days (Table 4), and five miRNAs were differentially expressed at four days of stress (Table 5). For five of these seven miRNAs, the differential expression profiles obtained by sequencing displayed the same expression profiles by RT-qPCR (Fig. 7): ssp-miR164, ssp-miR394, ssp-miR397, ssp-miR399-seq 1 and ssp-miR528. The lack of correlation observed for the other two miRNAs is likely due to cross-amplification of miRNA variants or to the presence of very similar miRNAs in the RT-qPCR samples.

The putative targets of the miRNA modulated by drought provide interesting clues to the drought response in sugarcane. For instance, a NAC transcription factor was found among the targets of ssp-miR164 (Table 6), in agreement with previous studies [44], [45]. It is known that miR164 regulates the expression of five NAM/ATAF/CUC (NAC) proteins in *Arabidopsis*. This class of plant-specific transcription factors has important roles in development, growth and stress responses, such as cold, drought and pathogen attach [46], [47], . In one study, overexpressing a member of the NAC gene family resulted in enhanced salt tolerance in tobacco [51]. In addition, NAC is responsible for transmitting auxin signals [52]. Another ssp-miR164 target was an MDR-like ABC transporter that also participates in auxin transport [53]. In this study, ssp-miR164 was differentially expressed in response to drought after only two days, suggesting that this miRNA acts in the early stage of the stress response. Considering that the targets of this miRNA presumably help sugarcane plants to withstand drought stress, we would expect that ssp-miR164 targets are repressed in both cultivars. This was the case for the LT cultivar (Fig. 6 and 7), suggesting that both NAC and the MDR-like ABC transporter, and possibly auxin, play roles in the drought response. However, in the HT cultivar, ssp-miR164 was induced by drought. These data indicate that the two analyzed cultivars differ in their activation of molecular mechanisms in response to drought and that the target genes may play a more relevant role in the LT cultivar.

It is known that miR393 is commonly up-regulated during drought stress in *Arabidopsis*, *Oryza sativa*, M*edicago truncatula* and *Pinguicula vulgaris* [54]. In this work, the ssp-miR393 miRNA was also up-regulated under drought stress in the LT cultivar after four days (Fig. 6). The predicted target of ssp-miR393 encodes a putative protein similar to TIR1, an auxin receptor in *Arabidopsis thaliana*. TIR1 recognized the 3-indol-acetic acid (AIA) and promote the degradation of the Aux/AIA repressor by a protein ubiquitin ligase that binds to a conserved domain of the repressor. This degradation releases the transcription of auxin-regulated genes [55]. Recently, the expression of *TIR1* was related with the response to inorganic phosphate (Pi) in roots of *Arabidopsis thaliana*, [56]. Our data points to an involvement of auxin in sugarcane responses to drought.

ssp-miR394 was down-regulated under drought stress in the two sugarcane cultivars (Fig. 6), indicating that this miRNA is important in the drought stress response independently of the plant genotype. The predicted target of ssp-miR394 is the gene encoding a glyceraldehyde-3-phosphate dehydrogenase (GAPDH); see Table 6. GAPDH catalyzes the oxidation of D-glyceraldehyde-3-P (D-G3P) to 3-phosphoglycerate (3-PGA) with the generation of NADPH in the sixth step of glycolysis [57]. Due to the increased need for available ATP and NADH_2_ under drought conditions, glycolysis is usually enhanced. This hypothesis is supported by the fact that rice plants exposed to drought display increased levels of *OsGAPDH* transcripts [58]. In this context, a decrease in the ssp-miR394 level would increase the GAPDH level and consequently the ATP content.

We also found that the target of ssp-miR397 is a gene encoding a laccase (Table 6), in agreement with previous studies [59]. Laccases are glycoproteins with roles in lignin synthesis [60] and iron acquisition [61]. It has been proposed that laccases are involved in cell wall modifications, such as lignification, acting to reduce cell wall extensibility and elongation [62], [63]. In maize [64] and tomato [65], increases in laccase transcripts were reported at high concentrations of NaCl. Under drought stress, ssp-miR397 was up-regulated in both cultivars on the second day (Fig. 6), suggesting a reduction in laccase expression levels. However, on the fourth day of drought stress, this miRNA was down-regulated in the HT cultivar (Fig. 6), likely leading to increased laccase levels, which would be reflected as a decrease in cell elongation due to lignin accumulation in the cell wall in response to stress. The complex expression pattern observed between the two cultivars indicates that lignification may act in different ways during drought stress.

miR399 has been described as a negative regulator of inorganic phosphate concentration because it targets a pyrophosphatase [66]. In this study, we also found an inorganic pyrophosphatase 2-like protein to be a target of ssp-miR399-seq 1 (Table 6). Overexpression of *AVP1 (Arabidopsis* vacuolar pyrophosphatase gene) results in increased drought and salt tolerance in transgenic *Arabidopsis*, tomato, rice and cotton plants [67], [68], [69], [70]. An increased proton pump activity by the vacuolar pyrophosphatase is the molecular explanation for drought resistance in these plants. This activity leads to a lower water potential in the plant vacuole and increases the activity of secondary transporters, preventing ion accumulation in the cytoplasm [70]. The down-regulation of ssp-miR399-seq 1 under drought stress in both sugarcane cultivars likely leads to increased pyrophosphatase levels. These data suggest that pyrophosphatases also play a role in sugarcane responses to drought stress and that this role is at least partially controlled by the microRNAs.

ssp-miR528 targets a gene encoding a UBX domain-containing protein (Table 6). UBX domains have been identified in several proteins with diverse functions in eukaryotes. Among the 15 UBX-containing proteins encoded by the *Arabidopsis* genome [71], PUX1 was shown to play important roles in plant growth and development. Loss-of-function mutant *pux1* plants display accelerated growth in various plant organs, including roots and inflorescence shoots [72]. In this work, ssp-miR528 was down-regulated under drought stress in both cultivars on the fourth day (Fig. 6), suggesting that this miRNA may be involved in the reduction of growth under this stress.

The expression levels of ssp-miR397, ssp-miR399-seq 1 and ssp-miR528 were quite variable in the control plants (Fig. 6), and this variability is likely due to other environmental conditions and/or intrinsic changes, such as developmental influences.

A gene encoding a bZIP transcription factor was determined to be an ssp-miR1432 target (Table 6). Interestingly, several bZIPs are known to play important roles in conferring stress tolerance to plants [73] by regulating the expression of genes that are involved in mechanisms that are essential for stress adaptation, such as cytoplasmic ion homeostasis and osmotic adjustment [74]. A triple knockout in the genes encoding the bZIPs AREB1, AREB2, and ABF3 in *Arabidopsis* displayed increased tolerance to ABA and reduced drought tolerance [75]. ssp-miR1432 was down-regulated by drought at similar levels at all time points in both cultivars (Fig. 6), suggesting that the sugarcane bZIP activates genes that help sugarcane plants to overcome drought stress. However, because the expression patterns observed in both cultivars are similar, this transcription factor alone may not be responsible for the different levels of drought tolerance in RB867515 and RB855536.

It is notable that the expression patterns of the majority of the miRNAs did not display clear correlations with the differences in drought tolerance observed in the two sugarcane cultivars. Nevertheless, it is clear that the microRNAs differ considerably between the two cultivars, suggesting that the miRNAs with differential expression do not fully explain the drought higher tolerance observed in RB867515.

In summary, our results provide insight into the sugarcane microRNAs, highlighting the regulatory network triggered by drought stress in an important bioenergy crop. We found that transcription factors, kinases, phosphatases and chaperones may be targets of the miRNAs modulated by drought in sugarcane. Further work with transgenic sugarcane overexpressing or silencing the miRNAs or their targets will increase our knowledge of the molecular mechanism of drought stress response and tolerance in sugarcane.

## Materials and Methods

### Plant samples

Sugarcane cultivars RB867515 (high tolerance to drought) and RB855536 (low tolerance to drought) were obtained from RIDESA (Rede Universitária para o Desenvolvimento do Setor Sucroalcooleiro). Plants were grown in a greenhouse at the Federal University of Alagoas (Alagoas, Brazil, 9°45′32″S, 36°13′09″W) under normal irrigation for three months. To develop drought stress, water was withheld from the test plants. The rehydrated plants received normal watering for two days after the period without irrigation. The control plants received normal watering throughout the experiment. Tissue was collected from Leaf +1 (the highest unfolded leaf with a visible dewlap) in quadruplicate after 2, 4, 6 and 8 days from irrigated, non-irrigated (drought-stressed) and rehydrated plants. The samples were quick-frozen and maintained at −80°C until RNA extraction using the miRVana miRNA isolation kit (Life Technologies, USA) according to the manufacturer's protocol. A pool of two replicates was used for Solexa sequencing, whereas four individual plants were used for RT-qPCR validation.

### Small RNA Sequencing

RNAs from 16 to 27 nt long were selected by polyacrylamide gel electrophoresis, ligated with adaptors at both ends and the products used for cDNA synthesis at BGI (Beijing Genomic Institute, Tai Po, Hong Kong). The sequencing was performed using the Solexa platform at BGI.

### Bioinformatics analysis

To identify the sequences corresponding to true mature miRNAs, we predicted their precursors (pre-miRNAs). Reads were preprocessed by removing adapters and discarding reads <18 nt. The miRDeep-P program [21] was used to map the reads to the reference sequences (*Sorghum bicolor* genomic sequence and sugarcane ESTs). For a given mapped read, a 250 bp window was used from which reference sequences were extracted to predict RNA secondary structure [76]. The miRDeep core algorithm with a plant-specific scoring system based on known characteristics of plant miRNA genes was used to determine the secondary structure of the extracted reference sequences [35]. RNA sequences were classified as miRNA precursor candidates based on the following parameters: the folding of the RNA sequence into a characteristic stem-loop hairpin secondary structure, the position of the mature miRNA in one arm of the hairpin structure (allowing a maximum of six mismatches with the miRNA* sequence in the opposite arm), negative MFE values for the predicted secondary structures, and a G/C content in the 30–70% range [38], using the RNAfold [77]. After the normalization of the number of reads in each library, the expression of each miRNA was calculated using the Audic-Claverie method [78].

The targets of the miRNA were predicted using psRNATarget (http://plantgrn.noble.org/psRNATarget/) by searching for target genes based on both complementarity scoring and secondary structure analyses [79]. The BlastX algorithm [80] was used to find the hits in the Sugarcane Assembled Sequences (SAS) against the NCBI database to identify the coding strands because several SASs corresponded to the minus strand. Only hits complementary to the coding strand of the sugarcane mRNAs were selected.

### Stem-loop reverse transcription and RT-qPCR validation

RT-loop primers (loop-RT), sequence-specific forward PCR primers (loop-FW) and universal reverse primers were designed following Chen et al., 2005 [30] for reverse transcription and PCR amplification of sugarcane miRNAs (Table S2). Reverse transcriptase reactions and cDNA amplification were performed as described by Varkonyi-Gasic et al., 2007 [31]. The miRNA and reference gene reverse transcriptase reactions were conducted under the same reaction condition.

### RT-qPCR miRNA and target validation

To validate and quantitate the expression levels of the miRNAs in sugarcane leaf tissues, RT-qPCR was performed using SYBR Green PCR Master Mix (Life Technologies, USA) on a 7500 Real-Time PCR System (Life technologies, USA). Each PCR reaction (18 µL) included 2 µL cDNA, 10 µL SYBR Green Master Mix (1×), 1 µL sequence-specific forward primer (10 µM), 1 µL universal reverse primer (10 µM) and 4 µL water. The sugarcane polyubiquitin gene [81] was used as a reference (Table 7). The reactions were performed at 95°C for 10 minutes followed by 40 cycles of 95°C for 15 seconds and 60°C for 1 minute, with a final dissociation curve analysis. All reactions were run with four biological replicates, each in triplicate. The real-time PCR data analysis was performed by considering the reaction efficiencies to calculate the fold changes in miRNA levels using a web-based QPCR system [82].

For target validation, each reverse transcriptase reaction contained 2.5 µg of total DNA-free RNA, 1 µL oligo d(T)_17_VN (50 µM) and 1 µL of dNTPs mix (10 µM). The reaction was incubated for 10 minutes at 65°C and then laid on ice for 2 minutes. Subsequently, 5× First Strand Buffer, DTT, RNAseOut and Superscript II enzyme (Life technologies, USA) were added. This reaction was incubated in a VerityTM Thermal Cycler (Applied Biosystems, USA) for 30 minutes at 42°C, followed by 15 minutes at 70°C. Forward and reverse specific PCR primers (Supporting Information, Table S2) were designed for amplification of the target genes. The reactions were carried out using the SYBRGreen PCR Master Mix (Applied Biosystems, USA) on 7500 Real Time PCR System (Applied Biosystems, USA) as described before.

## Supporting Information

Text S1 **Supporting information text.** Physiological data.
(DOCX)

Figure S1 **Quantum potential efficiency (Fv/Fm) in sugarcane plants.** The sugarcane cultivars RB855536 (LT - lower drought tolerance) and RB867515 (HT- higher drought tolerance) were maintained under irrigation, without irrigation and without irrigation and then rehydrated, as indicated. * indicates differences between irrigated and drought-stressed plants; +indicates differences between irrigated and rehydrated plants.** and ++indicate p<0.01, and * and +indicate p<0.05 using the t-test. ns - not significant.
(PNG)

Figure S2 **Fluorescence quantum yield (ΦPSII) in sugarcane plants.** The sugarcane cultivars RB855536 (LT - lower drought tolerance) and RB867515 (HT- higher drought tolerance) were maintained under irrigation, without irrigation and without irrigation and then rehydrated, as indicated. * indicates differences between irrigated and drought-stressed plants; +indicates differences between irrigated and rehydrated plants. ** and ++indicate p<0.01, and * and +indicate p<0.05 using the t-test. ns - not significant.
(PNG)

Figure S3 **Expression profile of the predicted target genes for the seven sugarcane miRNAs modulated by drought.** The values are expressed as fold changes relative to the irrigated control for each gene. The bars represent the average of the irrigated plants (control, black bars) and drought-stressed plants (gray bars) for RB867515 (HT) and RB855536 (LT) after two (2) and four (4) days of stress: A) HT2; B) HT4; C) LT2; D) LT4. Error bars represent the standard deviation (n = 3). Statistics was calculated between irrigated and drought treatments using the t- test. * indicates differences between irrigated and drought-stressed plants, with p≤0.05.
(PNG)

Table S1 **Target prediction of the miRNAs differentially expressed in drought-stressed sugarcane plants.** (All bioinformatics data without selection.) Target Acc: accession number in the SUCEST or SoGI databases; Expectation: value assigned to the alignment of the mature miRNA and the target, ranging from 0 (perfect alignment) to 5; UPE: the energy required to open the secondary structure of the target at the site recognition (less energy means better accessibility to the target); Mature miRNA: miRNA mature size (in nucleotides); Target start: the base position where the annealing with the miRNA starts; Target end: the base position where the annealing with the miRNA ends; Inhibition: the type of regulation by the miRNA; and Target description: description of the target according to a BLAST search in the GenBank database, including the name of the organism presenting the best hit.
(PNG)

Table S2 **Primers used in the reverse transcription and real-time PCR analyses of sugarcane miRNAs and target genes expression.** RT: primer loop for reverse transcription; FW: forward primer for real-time PCR; Rv: reverse primer for real-time PCR; reverse universal: reverse primer for miRNA real-time PCR; PUB: polyubiquitin gene primer; tgt: target gene; the complete sequence of each primer is shown and also the number of nucleotides (Nt) and the melting temperature (Tm) in °C.
(PNG)
