# Supplementary material for: microRNAs Associated with Drought Response in the Bioenergy Crop Sugarcane (Saccharum spp.)
Source: PLoS One. 2012 Oct 11;7(10):e46703. doi: 10.1371/journal.pone.0046703 (PMC3469577; doi:10.1371/journal.pone.0046703)
Supplement: Text S1 — Supporting information text. Physiological data. (DOCX) [file pone.0046703.s001.docx]

**Supporting Information text**

Sugarcane plants were adapted in the dark for 20 min, and a portable fluorometer (Opti-Sciences model OS1-FL, Hudson, USA) was used to measure the minimum fluorescence yield (F_0_), maximum fluorescence yield (Fm) and the variable fluorescence (Fv). The Fv/Fm ratio, indicating the light-induced chlorophyll fluorescence, represents the Photosystem II activity and was used to evaluate the damage caused by drought to the photosynthetic efficiency of the sugarcane plants.

When chlorophyll fluorescence was evaluated in the control, water-stressed and rehydrated plants (see Materials and Methods), photoinhibition increased over the period of drought stress, leading to strong reductions in Fv/Fm in both cultivars (Figure S1). The HT cultivar RB867515, when rehydrated, displayed a higher capacity to recover the Fv/Fm ratio, as expected.

The fluorescence quantum yield, ΦPSII, is an indicator of the light used in photochemistry by the chlorophyll associated with the PSII. This parameter was also negatively affected in both cultivars during drought stress (Figure S2). LT plants were able to recover only a fraction of their original ΦPSII levels upon rehydration, whereas ΦPSII in the HT plants fully recovered to the levels observed in the control plants.
