# Supplementary figures and images for: microRNAs Associated with Drought Response in the Bioenergy Crop Sugarcane (Saccharum spp.)
Source: PLoS One. 2012 Oct 11;7(10):e46703. doi: 10.1371/journal.pone.0046703 (PMC3469577; doi:10.1371/journal.pone.0046703)

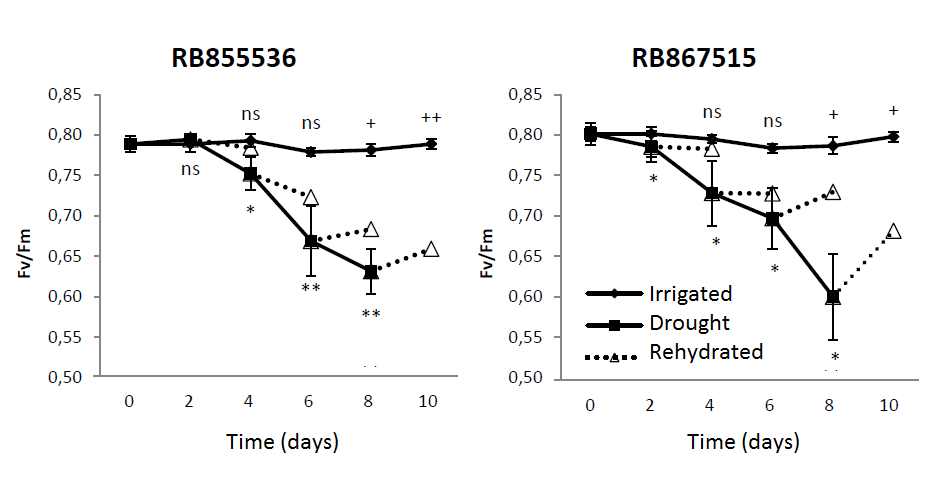

Supplement: Figure S1 — Quantum potential efficiency (Fv/Fm) in sugarcane plants. The sugarcane cultivars RB855536 (LT - lower drought tolerance) and RB867515 (HT- higher drought tolerance) were maintained under irrigation, without irrigation and without irrigation and then rehydrated, as indicated. * indicates differences between irrigated and drought-stressed plants; +indicates differences between irrigated and rehydrated plants.** and ++indicate p<0.01, and * and +indicate p<0.05 using the t-test. ns - not significant. (PNG) [file pone.0046703.s002.png]

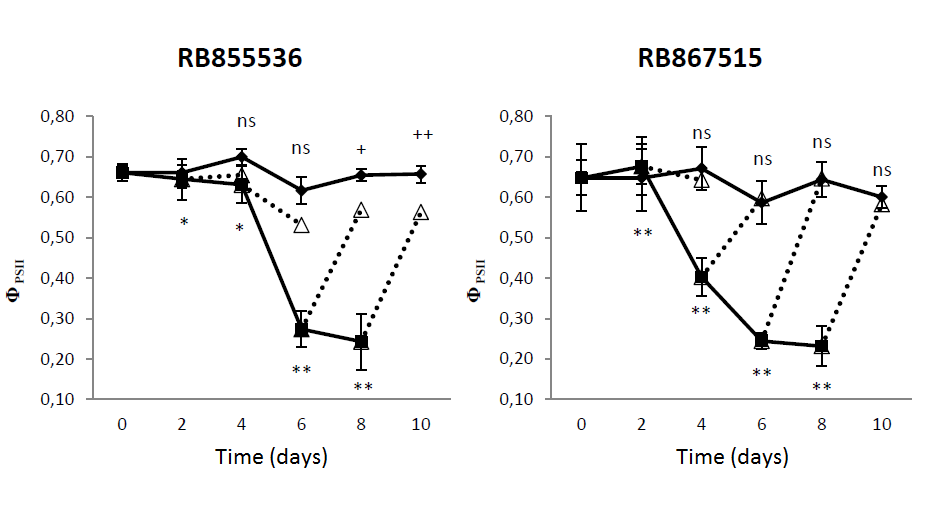

Supplement: Figure S2 — Fluorescence quantum yield (ΦPSII) in sugarcane plants. The sugarcane cultivars RB855536 (LT - lower drought tolerance) and RB867515 (HT- higher drought tolerance) were maintained under irrigation, without irrigation and without irrigation and then rehydrated, as indicated. * indicates differences between irrigated and drought-stressed plants; +indicates differences between irrigated and rehydrated plants. ** and ++indicate p<0.01, and * and +indicate p<0.05 using the t-test. ns - not significant. (PNG) [file pone.0046703.s003.png]

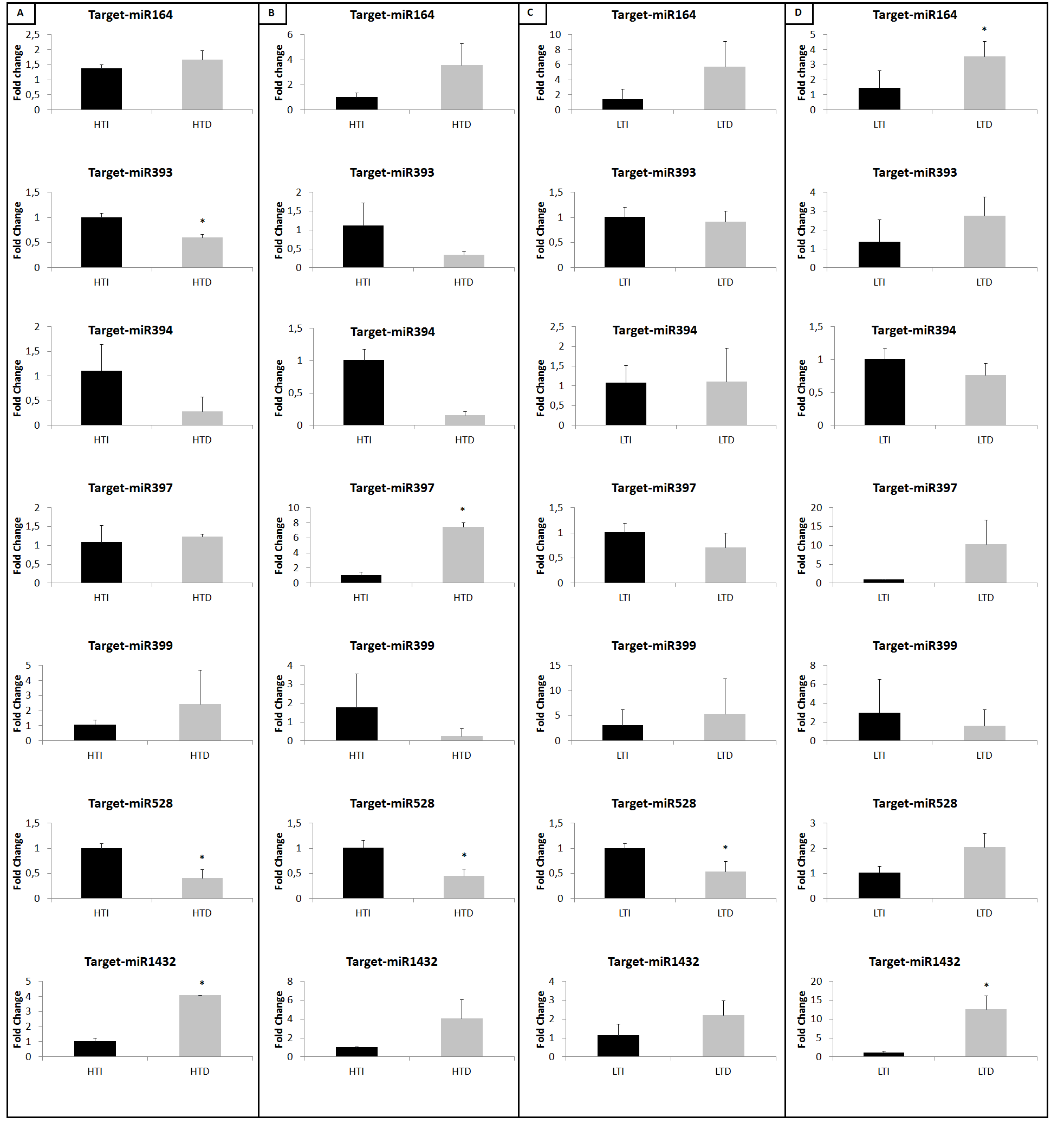

Supplement: Figure S3 — Expression profile of the predicted target genes for the seven sugarcane miRNAs modulated by drought. The values are expressed as fold changes relative to the irrigated control for each gene. The bars represent the average of the irrigated plants (control, black bars) and drought-stressed plants (gray bars) for RB867515 (HT) and RB855536 (LT) after two (2) and four (4) days of stress: A) HT2; B) HT4; C) LT2; D) LT4. Error bars represent the standard deviation (n = 3). Statistics was calculated between irrigated and drought treatments using the t- test. * indicates differences between irrigated and drought-stressed plants, with p≤0.05. (PNG) [file pone.0046703.s004.png]

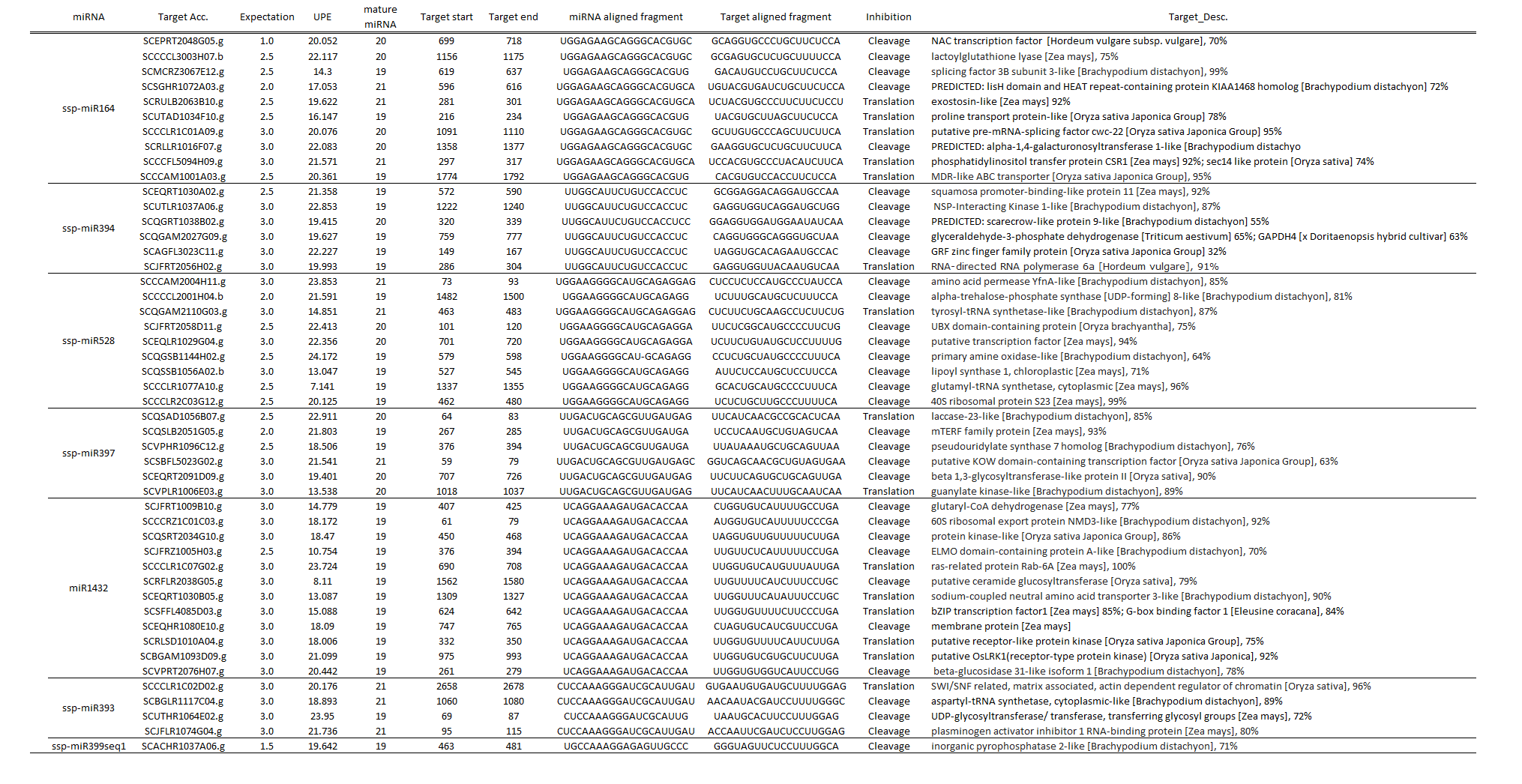

Supplement: Table S1 — Target prediction of the miRNAs differentially expressed in drought-stressed sugarcane plants. (All bioinformatics data without selection.) Target Acc: accession number in the SUCEST or SoGI databases; Expectation: value assigned to the alignment of the mature miRNA and the target, ranging from 0 (perfect alignment) to 5; UPE: the energy required to open the secondary structure of the target at the site recognition (less energy means better accessibility to the target); Mature miRNA: miRNA mature size (in nucleotides); Target start: the base position where the annealing with the miRNA starts; Target end: the base position where the annealing with the miRNA ends; Inhibition: the type of regulation by the miRNA; and Target description: description of the target according to a BLAST search in the GenBank database, including the name of the organism presenting the best hit. (PNG) [file pone.0046703.s005.png]

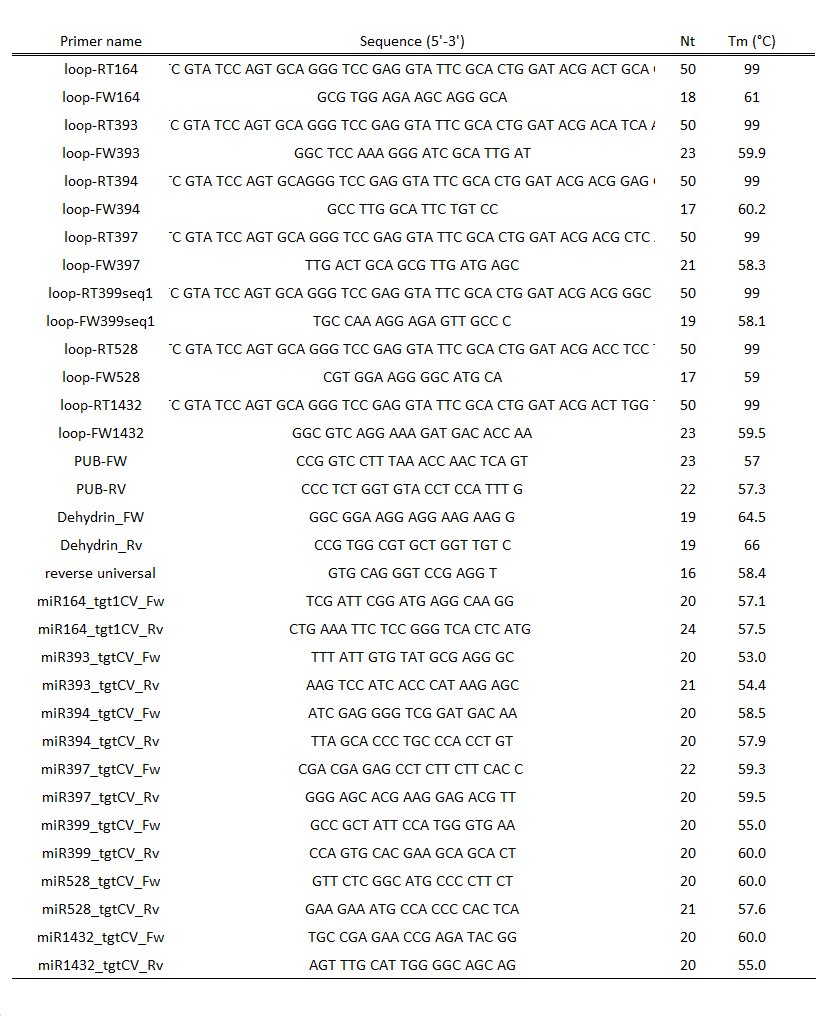

Supplement: Table S2 — Primers used in the reverse transcription and real-time PCR analyses of sugarcane miRNAs and target genes expression. RT: primer loop for reverse transcription; FW: forward primer for real-time PCR; Rv: reverse primer for real-time PCR; reverse universal: reverse primer for miRNA real-time PCR; PUB: polyubiquitin gene primer; tgt: target gene; the complete sequence of each primer is shown and also the number of nucleotides (Nt) and the melting temperature (Tm) in °C. (PNG) [file pone.0046703.s006.png]
